# High‐frequency ultrasound features of pemphigoid nodularis: A case report

**DOI:** 10.1111/srt.13077

**Published:** 2021-09-16

**Authors:** Xiaopo Wang, Jianfang Sun

**Affiliations:** ^1^ Department of Pathology Chinese Academy of Medical Sciences & Peking Union Medical College Hospital of Skin Diseases and Institute of Dermatology Nanjing China

## CONFLICTS OF INTEREST

None declared.

**FIGURE 1:**
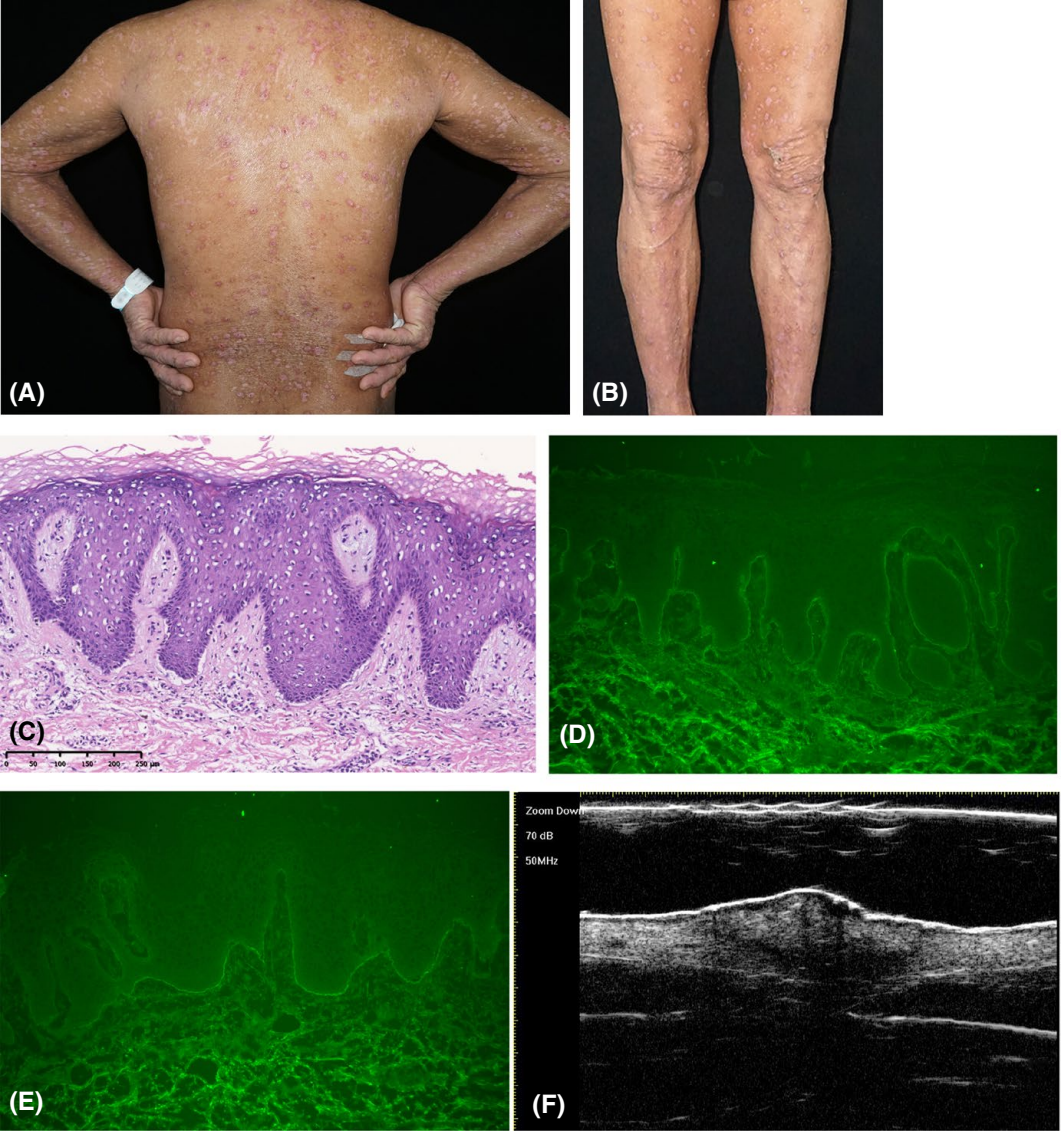
(A, B) Numerous discrete, excoriated, and erythematous nodules affecting the trunk and limbs; (C) A minimal subepidermal cleft with mild hyperkeratosis, epidermal hyperplasia, and perivascular lymphocytes infiltration (HE×100); (D, E) Direct immunofluorescence was positive for linear deposits of IgG and C3 along the basal membrane; (F) A well‐defined anechoic subepidermal cystic structures with a hypoechoic subjacent upper dermis with a 50‐MHz transducer

Dear Editor,

A 52‐year‐old otherwise healthy man presented with an 8‐month history of a nodular‐excoriated eruption on his trunk and extremities. He was previously diagnosed with prurigo nodularis in local hospital, and the treatment of oral antihistamines and potent topical corticosteroids resulted in limited benefit. Physical examination found numerous discrete, excoriated, and erythematous nodules affecting the trunk and limbs (Figure 1A,B). There were neither vesiculo‐bullous lesions nor mucous membrane involvement. The results of routine laboratory tests were unremarkable. A biopsy showed a minimal subepidermal cleft with mild hyperkeratosis, epidermal hyperplasia, and perivascular lymphocytes infiltration (Figure 1C). Direct immunofluorescence was positive for linear deposits of IgG and C3 along the basal membrane (Figure 1D,E). Enzyme‐linked immunosorbent assay from serum was positive for BP180 antibody (113.0 U/mL, normal range<9.0 U/mL) and was negative for BP230. In addition, a sonogram over the nodular eruption revealed well‐defined anechoic subepidermal cystic structures with a hypoechoic subjacent upper dermis with a 50‐MHz transducer (Figure 1F), which correlated histologically with subepidermal cleft and dermal inflammatory infiltrates. These findings were diagnostic for PN. The patient was then prescribed a combination of tripterygium wilfordii and a tapering dose of systemic corticosteroids for three months with dramatic improvement in the lesions and his symptoms.

Bullous pemphigoid is the most frequent autoimmune subepidermal blistering disease. Many atypical variants of bullous pemphigoid have been described, such as eczematous, urticarial, polycyclic, targetoid, nodular, lichenoid, or erythroderma.^1^ Pemphigoid nodularis (PN) is a rare form of bullous pemphigoid, characterized by clinical features of prurigo nodularis‐like lesions with an autoantibody profile of pemphigoid.^2^ For some patients, the correct diagnosis of PN is challenging because the blisters may be inconspicuous throughout the whole course of the condition. Histologic evaluation and immunofluorescence are needed to confirm the diagnosis.

Ultrasonography is a method of imaging that is classically used in dermatology to study changes in the subcutaneous tissue and deep structures. The high‐frequency ultrasound (HFUS) with little penetration and excellent resolution can be capable of clearly defining the superficial layer of the skin,^3^ which has been used to evaluate several types of skin disorders, such as skin cancer, some inflammatory and infectious cutaneous diseases.^4^, ^5^ Recently, there are some studies about the use of HFUS for assessing patients with bullous pemphigoid, which showed a well‐defined anechoic subepidermal cystic structure with a hypoechoic subjacent upper dermis.^6^, ^7^ However, to our knowledge, images of PN have not been described previously. The HFUS features of our patient with PN were similar to the bullous pemphigoid and have a high correlation with histologic findings.

Thus, HFUS may play an important role as an additional in vivo diagnostic tool to distinguish PN from prurigo nodularis.

In summary, HFUS detection of subepidermal cystic structures with dermal hypoechogenicity over a nodular eruption should raise suspicion of PN. While histology and immunofluorescence remain the gold standard for diagnosing PN, HFUS could provide a noninvasive tool to support clinicians in their decision to biopsy a nodular eruption.

## Data Availability

Data sharing is not applicable to this article as no new data were created or analyzed in this study.
